# Early Life Food Desert Status Is Associated With Alpha and Gamma‐Tocopherol Levels and Infant Lung Function

**DOI:** 10.1002/ppul.71479

**Published:** 2026-01-29

**Authors:** Garen S. Wolff, Angar Tsoggerel, Aki Hoji, Sydney E. Ross, Jay Colbert, Unai Miguel Andres, James E. Slaven, Joan Cook‐Mills, Kirsten M. Kloepfer

**Affiliations:** ^1^ Department of Pediatrics Division of Pulmonary, Allergy and Sleep Medicine, Indiana University School of Medicine Indianapolis Indiana USA; ^2^ Department of Pediatrics and Microbiology and Immunology Herman B. Wells Center for Pediatric Research, Indiana University School of Medicine Indianapolis Indiana USA; ^3^ The Polis Center, Luddy School of Informatics, Computing and Engineering Indiana University Purdue University Indianapolis Indianapolis Indiana USA; ^4^ Department of Pediatrics Division of Children's Health Services Research, Indiana University School of Medicine Indianapolis Indiana USA; ^5^ Department of Biostatistics and Health Data Science Indiana University School of Medicine and Indiana Clinical and Translational Sciences Institute Indianapolis Indiana USA

**Keywords:** alpha‐tocopherol, food deserts, health disparities, pulmonary function, Vitamin E

## Abstract

**Background:**

Living in a food desert (an area with limited access to affordable and nutritious food) is associated with a higher prevalence of childhood asthma. There is a lack of information regarding the impact of spending the first year of life in a food desert on subsets of Vitamin E (α‐ and γ‐tocopherol) levels and lung development.

**Objective:**

Determine if living in a food desert at 3 months of life is associated with altered α‐ and γ‐tocopherol, and infant lung measurements.

**Design:**

Newborns recruited within 1 week of delivery and prospectively followed for 3 months. 32 infants had sedated lung function tests and 50 had food desert data for analysis along with serum for α‐tocopherol and γ‐tocopherol analysis.

**Participants:**

Fifty (50) infants within the prospective Indiana High‐risk for Atopy in Neonates Cohort through Early life (INHANCE) were analyzed.

**Main Outcome Measures:**

Lung function, serum tocopherol concentration, and food desert status from the INHANCE cohort were analyzed. Because α‐tocopherol and γ‐tocopherol have opposing mechanistic functions, and the combination of high α‐tocopherol with low γ‐tocopherol have been shown to associate with better lung function in 2‐ to 3‐year olds and in adults, in this study of 3‐month old infants, quadrants of high and low α‐tocopherol and γ‐tocopherol were assessed for association with food deserts and lung function tests.

**Statistical Analyses Performed:**

Fisher's Exact tests were used to compare food desert designations with quadrants, due to small counts. Analysis of Variance (ANOVA) models were used to compare lung function values across the four quadrants, and Student's *t*‐tests were used to compare the lung function *z*‐scores across the two‐level quadrant groups.

**Results:**

At 3 months of age, lung volumes were lower in children living in food deserts (FVC: *p* = 0.006; FEV_0.5_: *p* = 0.008). None of the infants (*n* = 50) with the ideal tocopherol combination lived in a food desert compared to the other three quadrants with less ideal tocopherol combinations (*p* = 0.04). The infants (*n* = 32) with the ideal tocopherol combination had higher FRC (*p* = 0.006) and FEV_0.5_ (*p* = 0.025) *z*‐scores than infants in the other three quadrants.

**Conclusion:**

Not living in a food desert is associated with the highest α‐ and lowest γ‐tocopherol levels at 3 months of age. At 3 months of age, not living in a food desert was associated with higher lung function; with higher lung function associated with the highest α‐tocopherol and lowest γ‐tocopherol levels. Prospective trials are needed to determine if a lack of nutritious food during pregnancy and the first year of life is linked with decreased α‐tocopherol and increased γ‐tocopherol throughout this time period, and if this potential link is consistently associated with lower airway measurements that persist for the first few years of life.

**Abbreviations**
CARDIA: Coronary Artery Risk Development in Young Adults
FEF75: forced expiratory flow at 75% of vital capacity
FEF 50: forced expiratory flow at 50% of vital capacity
FEV0.5: forced expiratory volume in 0.5 s
FEV1: forced expiratory volume in 1 s
FRC: functional residual capacity
FVC: forced vital capacity
HPLC: high‐performance liquid chromatography
MCPHD: Marion County Public Health Department
SNAP: supplemental nutrition assistance program
USDA: United States Department of Agriculture
γ‐T: gamma‐tocopherol
α‐T: alpha‐tocopherol

## 1 Introduction

The United States Department of Agriculture (USDA) defines a food desert based on poverty rates, family income and distance to a grocery store [1]. In lieu of fresh food options like fruits, vegetables and whole grains provided by grocery stores, food deserts have a high density of fast‐food restaurants and convenience stores that provide processed foods high in sugar and fats [1].

The landscape of urban areas has a high percentage of both food deserts and racial and ethnic populations [2, 3, 4]. The burden of asthma in the United States falls disproportionately on these groups, resulting in high asthma rates, hospitalizations, and fatalities. Chronic diseases such as hypertension, diabetes, chronic kidney disease, and even pregnancy morbidity have been reported at higher rates in food desert inhabitants [5, 6, 7, 8]. However, a paucity of literature exists investigating the relationship between living in a food desert and asthma. At the time of this publication, the only study that exists was presented in abstract form at the American College of Allergy, Asthma, and Immunology in 2016 [9]. They found that living in a food desert correlated with an increased prevalence of pediatric asthma.

One way to measure ingestion of healthy food is by examining vitamin levels. Vitamin E is a fat‐soluble antioxidant that is composed of four isoforms of tocopherols: α‐tocopherol, γ‐tocopherol, β‐tocopherol and δ‐tocopherol. The isoforms, α‐tocopherol and γ‐tocopherol are the most abundant in our diet and are found in our tissues [10]. Olive and sunflower oils, along with breast milk, have high concentrations of α‐tocopherol. Canola and soybean oils, often used in processed foods, are rich in γ‐tocopherol and is the major form of tocopherol in the standard American diet. In mechanistic preclinical studies, α‐tocopherol reduces and γ‐tocopherol increases signals for recruitment of leukocytes during allergic lung inflammation in neonatal and adult mouse models [11]. In the past 40 years, an increase in γ‐tocopherol in both maternal diet and infant formula has paralleled the rise in asthma prevalence [10]. In the INSPIRE birth cohort in China, higher maternal postpartum plasma γ‐tocopherol was associated with recurrent wheezing in children in the first 2 years of life, suggesting that tocopherol plays a role in early airway inflammation [12]. Further investigations into the association between tocopherol and lung measurements in the Project Viva cohort revealed that in children with high α‐tocopherol plasma levels and low γ‐tocopherol at age 3 years had greater FEV_1_ at the mid‐childhood visit (age 6–10) compared to those who had high plasma γ‐tocopherol and low α‐tocopherol. This is consistent with opposing functions of tocopherol isoforms on lung function in children [11]. Furthermore, in the prospective study of adult participants (CARDIA cohort), higher levels of serum α‐tocopherol levels associated with higher FEV_1_ and FVC while higher γ‐tocopherol levels were associated with lower FEV_1_ or FVC by age 21 years. This was sustained up to age 55 years. Together, these studies suggest that a link between tocopherol isoforms and lung development is possible [13].

Based on these previous findings, we questioned if living in an area with limited access to α‐tocopherol enriched foods, such as a food desert, is associated with lower lung function during infancy. We hypothesized that not living in a food desert at 3 months of life is associated with higher lung function and increased serum levels of α‐tocopherol and decreased levels of γ‐tocopherol.

## 2 Methods

### 2.1 Database and Cohort Selection

This was a single‐center analysis of data on infants undergoing lung function testing from the Indiana High‐risk for Atopy in Neonates Cohort through Early‐life (INHANCE) cohort. Newborns were recruited within 1 week of delivery and prospectively followed for at least 3 months of life. Recruiting occurred between May 7, 2014–August 15, 2019. Over half of all mothers had a physician diagnosis of asthma (32/50). Fifty infants had food desert data for analysis and 32/50 had sedated lung function tests. All participants provided written informed consent. The study protocol (#1308055098) was approved by the Indiana University Human Subjects and Institutional Review Board.

### 2.2 Infant Lung Function Testing

Thirty‐two (32/50) infants underwent sedated infant lung function testing based on ATS/ERS standardized protocols using the Carefusion MasterScreen BabyBody (Hoechberg, Germany) [14]. A MD with sedation privileges cleared the infant before and after the procedure and was available throughout the procedure for questions/issues. Infants received 50 to 75 mg/kg of chloral hydrate orally from a trained BSN and measurements of forced expiratory flows were obtained while the infant was sleeping in the supine position. Raised volume rapid thoracoabdominal compression (RVRTC) lung function testing was performed by highly trained respiratory therapists with a combined 75 years of experience. Collectively, they have conducted well over 1000 infant pulmonary function tests, ensuring a high level of technical expertise and procedural consistency. Acceptable measurements were determined per ATS standards for repeatability (two trials with FVC, FEF_25–75_, and FEV_0.4/0.5_ within 10% of each other) [14]. The “best” trial was defined as the one with the highest sum of FVC and FEV_0.4/0.5_ or highest sum of FVC and FEF_25–75_. Measurements included: functional residual capacity (FRC), forced expiratory volume in 0.5 s (FEV_0.5_), forced vital capacity (FVC), forced expiratory flow at 50% and 75% of FVC (FEF_50_ and FEF_75_), and forced expiratory flows between 25% and 75% of FVC (FEF_25–75_). Predicted and standard deviation values were calculated for FVC, FEV_0.5_, FEF_25–75_, FEF_75_ [15], and functional residual capacity (FRC) [16]. To compare historical normative values, *z*‐scores were calculated for each value by subtracting the mean predicted value from the raw value and then dividing by the standard deviation [17]. Testing at age 3 months occurred prior to their first respiratory illness and their first diagnosis of wheeze.

### 2.3 Tocopherol Analysis

To examine if food desert at 3 months of life was related to changes in nutritional status, we examined associations with tocopherol isoform levels. This was chosen based on previous results from the CARDIA cohort in the United States that revealed that by age 21, higher levels of plasma α‐tocopherol is associated with better lung function and higher levels of plasma γ‐tocopherol associates with worse lung function [13]. Serum samples (*n* = 50) were taken from the INHANCE cohort at 3 months of age. Per previously published methods [11, 13] concentrations of tocopherol isoforms were determined by high‐performance liquid chromatography (HPLC). A detailed description of our analysis can be found in the eMethods section of this manuscript.

### 2.4 Food Desert Identification

An area that has either a poverty rate greater than or equal to 20% or a median family income not exceeding 80% of the median family income in urban areas, or 80% of the statewide median family income in nonurban areas may qualify as a food desert [1]. These data along with distance to a grocery store determines food desert designation. In urban areas, at least 500 people or 33% of the population must live more than 1 mile from the nearest large grocery store while in rural areas, at least 500 people or 33% of the population must live more than 10 miles from the nearest large grocery store [1]. To determine food desert status in our study, we converted parental addresses into coordinates to generate geocodes by, using the Polis Center standard geocoder. The resulting coordinates were intersected with block group data to determine if the children lived in a food desert. A detailed description of our analysis can be found in the eMethods section of this manuscript.

### 2.5 Statistical Analysis

Demographic characteristics were analyzed for both age groups using Student's *t*‐tests for continuous variables and Chi‐Square tests (or Fisher's Exact when 25% of responses had expected values < 5) for categorical variables. Fisher's Exact tests were used to compare food desert designations with quadrants, due to small counts. Analysis of Variance (ANOVA) models were used to compare lung function values across the four quadrants and Student's *t*‐tests were used to compare the lung function *z*‐scores across the two‐level quadrant groups. All analytic assumptions were verified, with transformations being used where appropriate (with raw scores being presented along with the *p*‐values from the transformed data comparisons). Analyses were performed using SAS v9.4 (SAS Institute, Cary, NC).

To analyze tocopherol isoform association with food desert and lung function, the median values for α‐tocopherol and median values for γ‐tocopherol were used to generate quadrants of tocopherol isoform levels at each age group. Tocopherol quadrant association with food deserts was assessed by the Fisher's Exact test. Tocopherol quadrant association with lung function tests was assessed by an ANOVA and Tukey's multiple comparison test. For comparisons to the ideal tocopherol quadrant (high α‐tocopherol, low γ‐tocopherol), an ANOVA and Dunnett test were utilized.

## 3 Results

### 3.1 Demographics

At 3 ± 2 months of age, 16/50 infants lived in a food desert. There was no difference in gestational age, method of delivery, sex at birth, cigarette smoke exposure, daycare attendance, or feeding choices (breastfed vs. formula) based on food desert status (Table 1).

**Table 1:** Demographics at 3 months of Age.

Maternal characteristics at birth	Food desert	Not food desert	*p* value
Age, mean (SEM), years	30.8 (1.37)	25.6 (0.92)	0.0135
History of asthma, *n* (%)	6/16 (37.5%)	26/34 (76.5%)	0.0074
Some college, *n* (%)	8/16 (50%)	18/34 (52.9%)	0.84
Private health insurance, *n* (%)	1/16 (6.3%)	7/33 (21.2%)	0.18
**Birth characteristics of infant**			
Gestational age, mean (SEM), weeks	38.8 (0.25)	38.7 (0.16)	0.57
Cesarean section, *n* (%)	2/16 (12.5%)	8/34 (23.5%)	0.36
Female, *n* (%)	9/16 (56.3%)	14/34 (41.2%)	0.31
Race, *n* (%)			0.39
Black	10 (63%)	16 (47%)	
White	3 (19%)	13 (38%)	
Asian	0	0	
Other	3 (18%)	5 (14.7%)	
Ethnicity, *n* (%)			
Hispanic/Latino	1 (6%)	8 (23.5%)	
Not Hispanic/Not Latino	15 (94%)	26 (76.5)	0.24
**Postnatal characteristics of infant**			
Caretaker smokes at 3 months	8/16 (50%)	12/34 (35.3%)	0.32
Breastfed at 3 months (%)	8/15 (53.3%)	12/31 (38.7%)	0.34
Pets in home in first year (%)	8/16 (50%)	15/34 (44.1%)	0.69
Eczema (%)	4/16 (25%)	13/34 (38.2%)	0.35
Food allergy (%)	0/16 (0%)	5/34 (14.7%)	n/a
Daycare	1/16 (6.3%)	4/34 (16.7%)	0.54

### 3.2 Tocopherol Levels

**Figure 1:**
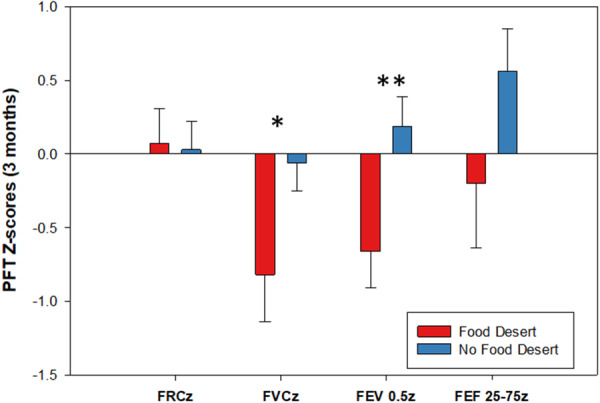
Pulmonary function testing z‐scores [18, 19] at 3 months of age based on food desert identification. (*p = 0.04; **p = 0.01). FRC = functional residual capacity, FEV_0.5_ = forced expiratory volume in 0.5 s, FVC = forced vital capacity, FEF_75_ = forced expiratory flow at 75% of FVC, and FEF_25–75_ = forced expiratory flows between 25% and 75% of FVC. [Color figure can be viewed at wileyonlinelibrary.com]

Fifty INHANCE participants had sufficient volumes of serum to assess tocopherol levels at 3‐months. Median γ‐tocopherol levels were 2.6 µM and α‐tocopherol was 28 µM. Because α‐tocopherol and γ‐tocopherol have opposing functions [10], the median tocopherol values were used to set quadrants of tocopherol isoform levels (Figure 1), as has been used in other studies with opposing functions of α‐tocopherol and γ‐tocopherol and 3‐yr old lung function [11]. Quadrants 1 (Q1), 2 (Q2) and 3 (Q3) are subjects with γ‐tocopherol:α‐tocopherol ratios that are higher than quadrant 4 (Q4). Q4 represents the ideal median for α‐tocopherol and γ‐tocopherol levels.

### 3.3 Food Deserts and Tocopherol Levels

At 3 months of age, 9/50 (18%) of study participants had the ideal tocopherol combination (high α‐tocopherol and low γ‐tocopherol, Q4); none of these infants lived in a food desert. All of the infants who lived in a food desert had α‐tocopherol and γ‐tocopherol levels within quadrants 1, 2 or 3 (Fisher's Exact test, *p* = 0.04). To further determine if α‐tocopherol and γ‐tocopherol levels were linked with residing in a food desert, we compared each quadrant separately. There was a trend for significance when comparing Q4 (high α‐tocopherol and low γ‐tocopherol) to those with low α‐tocopherol and high γ‐tocopherol (Q1), high α‐tocopherol and high γ‐tocopherol (Q2), and low α‐tocopherol and low γ‐tocopherol (Q3) (Fisher's Exact test, *p* = 0.06) (Table 2).

**Table 2:** Comparison of Food Desert Designation and quadrants of combinations of serum alpha‐tocopherol and gamma‐tocopherol levels.

3 months of age	Food desert	No food desert	*p* value
Comparison of 2 groups			
Q1, Q2 and Q3	16 (100)	25 (73.5)	0.042
High‐αT and low‐γT (Q4)	0 (0)	9 (26.5)	
Comparison of 4 groups			
Q1: low αT and high γT	2 (12.5)	6 (17.7)	0.06
Q2: high αT and high γT	6 (37.5)	11 (32.4)	
Q3: low αT and low γT	8 (50.0)	8 (23.5)	
Q4: High αT and low γT	0 (0)	9 (26.5)	

*Note:* Values are frequencies (percentages) with *p*‐value from Fisher's Exact test.

Abbreviations: αT, alpha‐tocopherol; γT, gamma‐tocopherol.

### 3.4 Food Deserts and Lung Function Testing

**Figure 2:**
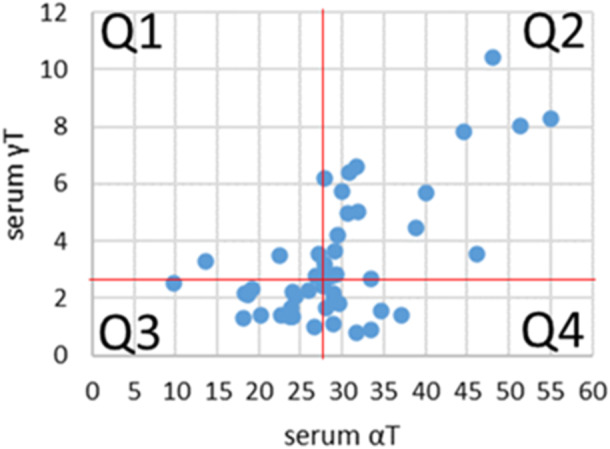
At 3 months of age, median gamma‐tocopherol level was 2.6 µM and alpha‐tocopherol was 28 µM. αT, alpha tocopherol; γT, gamma‐tocopherol. [Color figure can be viewed at wileyonlinelibrary.com]

Sedated lung volumes were measured at 3 months of age in 32/50 infants. On the day of lung function testing, median infant height was 61.9 cm ± 2.88 and weight was 6.36 kg ± 0.86. Infants not living in a food desert had higher FVC (−0.82 ± 1.09 vs. −0.06 ± 0.86, *p* = 0.04) and FEV_0.5_ (−0.66 ± 0.88 vs. 0.19 ± 0.88, *p* = 0.01) *z*‐scores (Figure 2). No difference was observed in FRC or FEF_25–75_.

Because food desert designation does not take into consideration the possibility that someone living in a rural environment may not have reliable transportation and cannot travel a few miles to the closest grocery store, we also examined if the number of miles to the closest grocery store was associated with lung function. An association between distance to grocery stores and lung function was not observed.

### 3.5 Tocopherol Levels and Lung Function

To determine if infant lung flow volumes in those who completed lung function testing were affected by α‐tocopherol and γ‐tocopherol levels, we compared the four quadrants separately finding the infants in Q4 had higher FRC (Q4 mean: 0.14 ± 0.78 vs. Q1‐3: −0.51 ± 0.16; *p* = 0.006) and FEV_0.5_ (Q4 mean: 0.62 ± 0.82 vs. Q1‐3: −0.21 ± 0.6; *p* = 0.025) *z*‐scores than infants in the other three quadrants (Table 3).

**Table 3:** 3‐month Infant PFTs (*n* = 32) in those with ideal vitamin E levels (Q4) and those with other combinations of alpha tocopherol and gamma tocopherol levels (Q1, Q2, Q3).

	Q1, Q2 and Q3 combined	Q4 (high alpha tocopherol and low gamma tocopherol)	*p* value
FRC *z*‐score	−0.51 (0.16)	0.14 (0.78)	0.0058
FVC *z*‐score	−0.25 (0.86)	0.25 (0.73)	0.26
FEV 0.5 *z*‐score	−0.21 (0.60)	0.62 (0.82)	0.0246
FEF 75 *z*‐score	−0.55 (1.77)	0.53 (1.15)	0.23
FEF 25–75 *z*‐score	0.06 (1.57)	1.05 (0.71)	0.19

Abbreviations: FEF_75_, forced expiratory flow at 75% of FVC; FEF_25–75_, forced expiratory flows between 25% and 75% of FVC; FEV_0.5_, forced expiratory volume in 0.5 s, FRC, functional residual capacity; FVC, forced vital capacity.

## 4 Discussion

To our knowledge, this is the first study to investigate the association between infant pulmonary function testing, food desert data, and tocopherol levels. In this study, we found that not living in a food desert is associated with the highest α‐tocopherol and lowest γ‐tocopherol levels at 3 months of age. This is consistent with previous data demonstrating that living in a food desert is associated with poor access to affordable, nutritious food [1]. Furthermore, at 3 months of age, a subset of infants living in food deserts had lower lung function values; with higher lung function values associated with the highest α‐tocopherol and lowest γ‐tocopherol levels. Based on these findings, we propose that high α‐tocopherol and low γ‐tocopherol levels are a result of easy access to healthy foods.

With the infant diet being limited between birth and 3 months of age, tocopherol levels would be influenced by maternal tocopherol dietary and supplement intake during pregnancy and breastfeeding, and by ingestion of tocopherol via oils in infant formula. A previous study reported high levels of α‐tocopherol in breastmilk after vitamin E supplementation [18] suggesting that this high level of α‐tocopherol is passed to the infant via breastmilk. However, there was not a difference in the number of breastfed infants in the food desert versus no food desert groups leading us to conclude that breastfed infants were not more likely to have ideal α‐tocopherol and γ‐tocopherol levels. Although this does not preclude that food deserts impact maternal tocopherol levels, as this needs further studies. Moreover, the type of formula did not correlate with food desert status with most of the formula fed infants living inside and outside of food deserts using either Gerber Good Start Gentle or Gerber Good Start Soothe. Our sample size was too small to determine if specific formulas were associated with ideal tocopherol levels, but formula is known to contain 5‐ to 18‐fold higher amount of γ‐tocopherol than human milk [19] so it is unlikely that formula brand would impact our results. Finally, a majority (67%) of mothers reported taking pre‐natal vitamins during pregnancy. We did not observe a difference in ingestion rates of prenatal vitamins between those living in a food desert and those not living in a food desert. As prenatal vitamins differ in levels of α‐tocopherol and γ‐tocopherol, it is possible that these played a role in infant serum tocopherol levels but without having the exact prenatal vitamin brand and tocopherol isoform levels, their impact cannot be determined.

It is possible that switching from breastmilk to formula at 3 months of age could alter plasma tocopherol composition. Previous data show that high α‐tocopherol and low γ‐tocopherol levels associate with higher lung function in infants and adults [13]. This higher lung function could be lowered if the infant is changed to a diet high in γ‐tocopherol. Perhaps environmental exposures begin to play a role in their developing airway, leading to complex interactions of multiple environmental factors that may impact lung function. Future studies should be designed to prospectively follow plasma tocopherol levels during pregnancy, in cord blood and prospectively during the first few years of life. Along with this, lung function and a detailed food and supplement diary should be recorded to help measure all sources of tocopherol.

Infant lung function testing in a subset of infants provides objective measurements that can be followed prospectively. Lower lung function values are linked with long term airway obstruction [20]. While we have measurements at 3 months, we did not have sufficient longitudinal lung measurements as these infants became children or adults. This prevents us from investigating long‐term sequalae from living in a food desert. Future studies should follow these infants long‐term to determine if this link persists, and to determine if inflammatory markers are associated with food desert status. A study designed to address changes in lung function as the infant ages as well as potential confounders such as maternal diet during pregnancy, illness, and environmental exposures is needed. One strength of our study was our ability to utilize complex computer analysis to determine food deserts. However, there are several influences on food accessibility that can be difficult to ascertain utilizing food desert data. These include accessibility to reliable transportation, cost of food, and store hours. Future studies should focus on these additional data to determine if they impact the airway.

A recent study from the Project Viva cohort found that vitamin E levels in maternal plasma during the second trimester did not correlate with childhood lung function values at ≥ 6 years of age [11]. However, in the same cohort, higher lung function measurements during school‐age (ages 6–10 years) were associated with the highest α‐tocopherol and lowest γ‐tocopherol levels at 3 years of age [11]. These data, along with our findings that the highest alpha‐tocopherol levels are associated with higher FEV_0.5_, suggests that if tocopherol levels influence lung function, the influence is short term and cannot predict long term lung function values. This is likely due to changes in diet during infancy and childhood with associated changes in α‐ and γ‐tocopherol levels. If tocopherol levels influence lung function, the length of time that the influence occurs is unknown. With our data only representing one point in time, our data are too limited to determine how quickly tocopherol levels change throughout the first year of life, and if these changes are consistently associated with changes in lung function.

Finally, we found that infants with high α‐tocopherol and low γ‐tocopherol levels have higher lung function measurements at 3 months of age. The decrease of FVC and FEV_0.5_ observed in this study can be consistent with airway obstruction but FEV_0.5_/FVC did not differ between the two groups making obstruction less likely. Administering a bronchodilator to measure reversibility would have been helpful to determine if any infants had obstruction but we do not have this data. Because we found an increase in FRC in those in Q4 along with an increase in FEV_0.5_, we question if our samples size was sufficient to draw these conclusions. It is possible that the high FRC is associated with incomplete exhalation during PFTs or inflammation leading to air trapping. Alongside the increase in FRC we also observed an increase in FEV_0.5_ which is not physiologically possible. Because we lack longitudinal data and inflammatory markers on these children, we cannot determine if airway inflammation is present. However, the elevated FRC and FEV_0.5_ increases the odds that our sample size is too small to make definitive conclusions on airway changes based on PFTs. Adding additional infants to future studies would help determine if airway inflammation is occurring.

## 5 Conclusion

In conclusion, this study adds to the evidence that not living in a food desert is associated with increased α‐tocopherol and decreased γ‐tocopherol and might be associated with altered lung function. Prospective trials are needed to determine if a lack of nutritious food during pregnancy and the first year of life is linked with decreased α‐tocopherol and increased γ‐tocopherol throughout this time period, and if this potential link is consistently associated with lower airway measurements that persist for the first few years of life.

## Author Contributions

Concept and design: Garen S. Wolff, Jay Colbert, Kirsten M. Kloepfer. Analysis and interpretation: Garen S. Wolff, Angar Tsoggerel, Aki Hoji, Sydney E. Ross, James E. Slaven, Jay Colbert, Unai Miguel Andres, Jay Colbert, Kirsten M. Kloepfer. Drafting of manuscript for important intellectual content: Garen S. Wolff, Jay Colbert, Kirsten M. Kloepfer.

## Conflicts of Interest

The authors declare no conflicts of interest.

## Supporting information

supmat.

## Data Availability

The data that support the findings of this study are available from the corresponding author upon reasonable request.

## References

[1] Service UER . Food Access Research Atlas (U.S. Department of Agriculture, 2023). https://www.ers.usda.gov/data-products/food-access-research-atlas/documentation/#:~:text=Definition%3A%20A%20tract%20in%20which,supermarket%2C%20regardless%20of%20vehicle%20availability.

[2] K. M. Bower , R. J. Thorpe, Jr. , C. Rohde , and D. J. Gaskin , “The Intersection of Neighborhood Racial Segregation, Poverty, and Urbanicity and Its Impact on Food Store Availability in the United States,” Preventive Medicine 58 (2014): 33–39. 24161713 10.1016/j.ypmed.2013.10.010 PMC3970577

[3] R. E. Walker , C. R. Keane , and J. G. Burke , “Disparities and Access to Healthy Food in the United States: A Review of Food Deserts Literature,” Health & Place 16, no. 5 (2010): 876–884. 20462784 10.1016/j.healthplace.2010.04.013

[4] N. I. Larson , M. T. Story , and M. C. Nelson , “Neighborhood Environments,” American Journal of Preventive Medicine 36, no. 1 (2009): 74–81.e10. 18977112 10.1016/j.amepre.2008.09.025

[5] J. J. Suarez , T. Isakova , C. A. M. Anderson , L. E. Boulware , M. Wolf , and J. J. Scialla , “Food Access, Chronic Kidney Disease, and Hypertension in the U.S,” American Journal of Preventive Medicine 49, no. 6 (2015): 912–920. 26590940 10.1016/j.amepre.2015.07.017 PMC4656149

[6] M. J. Tipton , S. A. Wagner , A. Dixon , L. Westbay , H. Darji , and S. Graziano , “Association of Living in a Food Desert With Pregnancy Morbidity,” Obstetrics & Gynecology 136, no. 1 (2020): 140–145. 32541293 10.1097/AOG.0000000000003868

[7] E. Gucciardi , M. Vahabi , N. Norris , J. P. Del Monte , and C. Farnum , “The Intersection Between Food Insecurity and Diabetes: A Review,” Current Nutrition Reports 3, no. 4 (2014): 324–332. 25383254 10.1007/s13668-014-0104-4 PMC4218969

[8] K. Morland , A. V. Diez Roux , and S. Wing , “Supermarkets, Other Food Stores, and Obesity,” American Journal of Preventive Medicine 30, no. 4 (2006): 333–339. 16530621 10.1016/j.amepre.2005.11.003

[9] D. M. M. Preston and A. Plunk , “The Relationship Between Asthma and Food Deserts in the Hampton Roads Area,” Annals of Allergy, Asthma & Immunology 117, no. 5 (2016): S8.

[10] J. M. Cook‐Mills , S. H. Averill , and J. D. Lajiness , “Asthma, Allergy and Vitamin E: Current and Future Perspectives,” Free Radical Biology and Medicine 179 (2022): 388–402. 34785320 10.1016/j.freeradbiomed.2021.10.037 PMC9109636

[11] R. Kumar , R. P. Ferrie , L. C. Balmert , et al., “Associations of α‐ and γ‐tocopherol During Early Life With Lung Function in Childhood,” Journal of Allergy and Clinical Immunology 146, no. 6 (2020): 1349–1357.e3 e3. 32344059 10.1016/j.jaci.2020.04.019 PMC7606217

[12] C. A. Stone, Jr. , J. Cook‐Mills , T. Gebretsadik , et al., “Delineation of the Individual Effects of Vitamin E Isoforms on Early Life Incident Wheezing,” Journal of Pediatrics 206 (2019): 156–163.e3 e3. 30527752 10.1016/j.jpeds.2018.10.045 PMC6415525

[13] M. E. Marchese , R. Kumar , L. A. Colangelo , et al., “The Vitamin E Isoforms α‐tocopherol and γ‐tocopherol Have Opposite Associations With Spirometric Parameters: The CARDIA Study,” Respiratory Research 15, no. 1 (2014): 31. 24629024 10.1186/1465-9921-15-31 PMC4003816

[14] S. Lum , J. Stocks , and R. Castile , “ATS/ERS Statement: Raised Volume Forced Expirations in Infants: Guidelines for Current Practice,” American Journal of Respiratory and Critical Care Medicine 172, no. 11 (2005): 1463–1471. 16301301 10.1164/rccm.200408-1141ST

[15] S. Lum , V. Bountziouka , A. Wade , et al., “New Reference Ranges for Interpreting Forced Expiratory Manoeuvres in Infants and Implications for Clinical Interpretation: A Multicentre Collaboration,” Thorax 71, no. 3 (2016): 276–283. 26526556 10.1136/thoraxjnl-2015-207278

[16] T. T. D. Nguyen , A. F. Hoo , S. Lum , A. Wade , L. P. Thia , and J. Stocks , “New Reference Equations to Improve Interpretation of Infant Lung Function,” Pediatric Pulmonology 48, no. 4 (2013): 370–380. 22949414 10.1002/ppul.22656

[17] J. A. Voynow , R. Feng , C. L. Ren , et al., “Pulmonary Function Tests in Extremely Low Gestational Age Infants at One Year of Age,” Pediatric Pulmonology 57, no. 2 (2022): 435–447. 34779149 10.1002/ppul.25757

[18] S. Gaur , M. J. Kuchan , C. S. Lai , S. K. Jensen , and C. L. Sherry , “Supplementation With RRR‐ or all‐rac‐α‐Tocopherol Differentially Affects the α‐Tocopherol Stereoisomer Profile in the Milk and Plasma of Lactating Women,” Journal of Nutrition 147, no. 7 (2017): 1301–1307. 28566525 10.3945/jn.116.245134

[19] D. Martysiak‐Żurowska , A. Szlagatys‐Sidorkiewicz , and M. Zagierski , “Concentrations of Alpha‐ and Gamma‐Tocopherols in Human Breast Milk During the First Months of Lactation and in Infant Formulas,” Maternal & Child Nutrition 9, no. 4 (2013): 473–482. 22513202 10.1111/j.1740-8709.2012.00401.x PMC6860560

[20] D. A. Stern , W. J. Morgan , A. L. Wright , S. Guerra , and F. D. Martinez , “Poor Airway Function in Early Infancy and Lung Function by Age 22 Years: A Non‐Selective Longitudinal Cohort Study,” Lancet 370, no. 9589 (2007): 758–764. 17765525 10.1016/S0140-6736(07)61379-8 PMC2831283

