# Supplementary material for: Early Life Food Desert Status Is Associated With Alpha and Gamma‐Tocopherol Levels and Infant Lung Function
Source: Pediatr Pulmonol. 2026 Jan 29;61(2):e71479. doi: 10.1002/ppul.71479 (PMC12853406; doi:10.1002/ppul.71479)
Supplement: Supplementary file 1 — supmat. [file PPUL-61-0-s001.docx]

eMethods

Tocopherol Analysis

Concentrations of tocopherol isoforms were determined by high-performance liquid chromatography (HPLC) with an electrochemical detector. The internal standard tocol (10uL of Tocol 25ug/mL), 25uL of serum sample, 50uL of 10% ascorbic acid in water, 1▒mL of 100% ethanol (HPLC-grade), 100uL of 0.2▒N Hydrochloric acid, and 1▒mL of hexane with 0.1% weight/volume butylated hydroxytoluene to prevent oxidation and increase recovery of tocopherol were combined in a borosilicate tube. The samples were vortexed and then centrifuged for 5▒minutes at (2000 g at 4°C) and the hexane layers were collected. The samples were extracted for a total of three times with addition of hexane/butylated hydroxytoluene. The hexane layers for each sample were combined and dried under nitrogen. The samples were reconstituted in methanol and the tocopherols were separated by using a reverse-phase C18 HPLC column (catalog No. WAT085711 [Waters Co, Milford, Mass]) and HPLC chromatography (Waters Co) with 99% methanol and 1% water as a mobile phase with detection with an electrochemical detector (potential 0.7▒V) (Waters Co). Standard curves containing tocol, α-T, γ-T, β-tocopherol, and δ-tocopherol (25-µL injection volume of 2.5, 1.25, 0.625, 0.312, 0.156, 0.078 and 0.039▒mg standards/mL) were analyzed with each sample run. Concentrations were calculated by using the area under the curve of the HPLC chromatograms, the standard curves, and tocol percent recovery from extractions. All samples were analyzed within the linear standard curve.

Food Desert Analysis

Drawing from the US Department of Agriculture, US Treasury and Health and Human Services (9), food desert census tract level definition, which identifies census tracts as Food Deserts if they are low income and low food access, we developed census Block Group level food desert layer using the following data and methodology.

Food retailer data was collected using the Marion County Public Health Department (MCPHD) food establishment inspections for Marion County and the USDA SNAP accepting retailers for the rest of the State of Indiana. Both sources included healthy food stores as well as pharmacies, gas stations, convenience stores and some restaurants. MCPHD data was requested and received via email; it can also be accessed through https://hhcwebfood.hhcorp.org/#. USDA retail data was retrieved from https://www.fns.usda.gov/snap/retailer-locator. After collecting the full retailer lists from both sources, the entries were verified to be healthy food stores (either a supermarket, grocery store, an ethnic grocer, or small grocer with items suitable for a healthy diet) using a combination of chain names, and verification of smaller and/or independent retailers via google maps, retail online sites and social media posts.

After narrowing down the list of food retailers to just healthy food stores, they were geocoded in ArcMap 10.5 using the Polis Center standard geocoder, a multi-address locator geocoder composed of street and point address locators housed on an Indiana University ArcGIS Server instance. The healthy food stores that were not automatically geocoded were manually geocoded until 100% of the retailers were located on the map and provided a latitude and longitude. The resulted geocoded file was then imported into ArcGIS Online. Once imported, road network service areas were created using the ArcGIS Online Create Drive-Time Areas tool. The tool was set to calculate two driving distances (1 & 10 Miles) away from the stores. The option of driving away rather than towards the store was chosen because when the distance and therefore time to get home from the store is more relevant than the distance and time of going towards the store (due to perishability of items and the capacity of carrying a maximum amount of weight for those individuals that might not have access to a car). Driving was chosen as the method for calculating the distance because this method uses the whole road system rather than just a sidewalk or bus route. The resulting service area layers were then imported into ArcGIS Pro 2.4.

The Indiana parcel data was retrieved from the Indiana Data Harvest project. Using the property class codes, we extracted non-vacant residential parcels as well as commercial rental parcels in ArcGIS Pro 2.4. The resulting home parcels were spatially intersected with the 1- & 10-mile services areas. Since we did not have a way to identify which side of the parcel provided access to the residence, spatial intersection was chosen for the spatial selection. Therefore, we assumed that if even just one corner or side of the parcel touched or was within the service area of a grocery store then the whole residence was within a close distance to it. Fieldswere developed identify if a parcel intersected any of the 1-mile service areas and any of the 10-mile service areas. These fields were populated using Boolean values, a 1 was given if the parcel intersected at least 1 service area of the specified distance, and a 0 if it didn’t intersect any. Finally, a field called “All_parcels” was created and populated with a value of 1 for all the parcels. Parcels were then aggregated into 2010 Census block groups using the Spatial Join. The aggregation was set to be a one-to-one join, to ensure that all the join values (parcels) were aggregated into the target feature (block groups) they were within. The rule of merge was set to Sum, so each field would summarize the Boolean values within them to determine the number of parcels within the block group, the number of parcels within the block group that were within 1 mile of a grocery store, and the number of parcels within the block group that were within 10 miles of a grocery store. Using the urban places layer from the Census bureau, block groups were classified as either urban or rural block groups. If a block group was determined to be urban, then the 1-mile stores service areas were used to determined food access. If a block group was determined to be rural, then the 10-mile service area was used instead. Socio-demographic information from the 2019 American Community Standard (ACS) 5-year average was added to the block group and used to determine if a block group was low food access, if it was low income, and if it was a food desert. A low food access block group was determined to be one where at least 33% of the parcels were not within the service area of grocery store or a block group were at least 200 people had low food access (calculated by multiplying the percentage of parcels within the block group outside of a service area by the total population of the block group). A low-income block group was determined to be ones that have a high poverty rate (at least 20%) or median household income significantly lower than the region’s (80% or less of the State median or their respective Metropolitan Statistical Area’s median). If a block group had low food access and was low-income, it was classified as a food desert.
